# Inflammatory and mitochondrial gene expression data in GPER-deficient cardiomyocytes from male and female mice

**DOI:** 10.1016/j.dib.2016.11.057

**Published:** 2016-11-23

**Authors:** Hao Wang, Xuming Sun, Jeff Chou, Marina Lin, Carlos M. Ferrario, Gisele Zapata-Sudo, Leanne Groban

**Affiliations:** a Department of Anesthesiology, Wake Forest School of Medicine, Medical Center Blvd., Winston-Salem, NC 27157-1009, USA; b Internal Medicine/Molecular Medicine, Wake Forest School of Medicine, Medical Center Blvd., Winston-Salem, NC 27157, USA; c Public Health Sciences, Section on Biostatistical Sciences, Wake Forest School of Medicine, Medical Center Blvd., Winston-Salem, NC 27157, USA; d Department of Surgery, Wake Forest School of Medicine, Medical Center Blvd., Winston-Salem, NC 27157, USA; e Department of Internal Medicine/Nephrology, Wake Forest School of Medicine, Medical Center Blvd., Winston-Salem, NC 27157, USA; f Institute of Biomedical Sciences, Drug Development Program, Federal University of Rio de Janeiro, Brazil; g Cardiovascular Research Center, Wake Forest School of Medicine, Medical Center Blvd., Winston-Salem, NC 27157, USA; h Sticht Center on Aging, Wake Forest School of Medicine, Medical Center Blvd., Winston-Salem, NC 27157, USA

**Keywords:** Cardiomyocyte, GPER, Knockout, Microarray, Mitochondria, Inflammation

## Abstract

We previously showed that cardiomyocyte-specific G protein-coupled estrogen receptor (GPER) gene deletion leads to sex-specific adverse effects on cardiac structure and function; alterations which may be due to distinct differences in mitochondrial and inflammatory processes between sexes. Here, we provide the results of Gene Set Enrichment Analysis (GSEA) based on the DNA microarray data from GPER-knockout versus GPER-intact (intact) cardiomyocytes. This article contains complete data on the mitochondrial and inflammatory response-related gene expression changes that were significant in GPER knockout versus intact cardiomyocytes from adult male and female mice. The data are supplemental to our original research article “Cardiomyocyte-specific deletion of the G protein-coupled estrogen receptor (GPER) leads to left ventricular dysfunction and adverse remodeling: a sex-specific gene profiling” (Wang et al., 2016) [1]. Data have been deposited to the Gene Expression Omnibus (GEO) database repository with the dataset identifier GSE86843.

**Specifications Table**

**Table:** 

Subject area	*Biology*
More specific subject area	*Heart disease, gene knockdown*
Type of data	*Tables*
How data was acquired	*Microarray data in cardiomyocytes generated using Affymetrix GeneAtlas 3*′*-IVT Express Kit*
Data format	*Analyzed*
Experimental factors	*Comparison of inflammatory and mitochondrial gene expression profiles of GPER-deficient versus intact cardiomyocytes from male and female mice*
Experimental features	*RNA isolation, global gene expression analysis, and bioinformatics analyses using Gene Set Enrichment Analysis (GSEA) software*
Data source location	*Wake Forest School of Medicine, Winston-Salem, NC, USA*
Data accessibility	*Dataset is within this article and available in the Gene Expression Omnibus with accession number GEO:*GSE86843.

**Value of the data**

• This dataset provides the complete list of altered genes related to mitochondria and inflammatory response in GPER-knockout versus intact cardiomyocytes from mice of both sexes.
• May facilitate further research that reveals the pathophysiology for sex-specific differences in heart disease.
• May serve as a benchmark for comparison with data obtained from estrogen receptor (ER) α and ERβ cardiomyocyte-specific knockout mice for further insight into the functional roles of the estrogen receptors in the maintenance of cardiac structure and function.
• May stimulate further research on the clinical potential of targeting GPER in the treatment of heart disease and other age-related disorders, in which mitochondrial dysfunction and inflammation have central roles in the underlying pathophysiology.

## 1 Data

To examine the differences in the mitochondrial and inflammatory response gene expressions between GPER-knockout and intact cardiomyocytes, microarray data were loaded into GSEA 2.0.1 software using GSEA gene sets “MITOCHONDRION (including 314 genes)” and “HALLMARK_INFLAMMATORY_RESPONSE (including 193 genes)” [1,2]. The altered individual mitochondrial and inflammatory genes in GPER knockout versus intact cardiomyocytes from both sexes are presented in Table 1, Table 2, Table 3, Table 4.

## 2 Experimental design, materials and methods

### 2.1 Cardiomyocyte isolation from GPER KO and GPER-intact or wild-type mice

Mice at 18–20 weeks of age were injected i.p. with 200 µl heparin (Sagent Pharmaceutical Inc., Schaumburg, IL, 100 IU/mouse) 10 min prior to anesthesia with pentobarbital (Akorn Inc., Lake Forest, IL, 100 mg/kg body weight) by i.p. injection. Upon verification of deep anesthesia by the absence of response to tail/toe pinches, the heart was quickly removed and trimmed in an ice-cold, calcium-free perfusion buffer (126 mM NaCl, 4.4 mM KCl, 1 mM MgCl2, 4 mM NaHCO3, 10 mM HEPES, 11 mM glucose, 30 mM 2,3-butanedine monoxime [Sigma, St. Louis, MO], 5 mM taurine [Sigma], pH 7.35). The heart was then cannulated through the aorta on an Easycell System for Cardiomyocyte Isolation (Harvard Apparatus, Holliston, MA) and perfused at 37 °C with calcium-free perfusion buffer at a flow rate of 3 ml/min for 4–5 min until the effluent became clear. The heart was switched to digestion buffer (perfusion buffer plus 50 µM CaCl2 and 0.5 mg/ml collagenase II [Worthington Biochemical Corp., Freehold, NJ]), and perfused for 10–15 min at a flow rate of 4 ml/min until the heart was pale and flaccid. The heart was pulled from the cannula and the ventricles were transferred to a 60-mm sterile dish containing 5 ml of transfer buffer (perfusion buffer plus 0.1 mM CaCl2 and 2% bovine serum albumin [Sigma]) and cut into small pieces. The minced tissue was incubated in a 37 °C water bath for 10 min. The cell suspension was filtered through a 100-μm mesh cell strainer (BD Biosciences, San Jose, CA) to remove tissue debris and spun at 420 rpm at room temperature for 2 min. After removing the supernatant, cardiomyocytes were washed with 1 ml of PBS and centrifuged at 1500 rpm at 4 °C for 3 min. The cells were suspended in 1 ml of QIAzol (Qiagen Inc, Valencia, CA), mixed, and homogenized before storing at −80 °C.

### 2.2 DNA microarray assay

Total RNA was isolated from cardiomyocytes using the RNeasy Lipit Tissue Mini Kit (Qiagen Inc) and further purified using RNeasy MinElute Cleanup Kit (Qiagen Inc) followed by quality assessment on an Agilent 2100 bioanalyzer. Samples with RIN values >8.0 and a 260/280 ratio between 1.8 and 2.1 were carried forward for cRNA synthesis and hybridization to GeneAtlas MG-430 PM Array Strips (Affymetrix, Santa Clara, CA) following the manufacturer׳s recommended protocol [3]. Briefly, approximately 250 ng of purified total RNA was reverse transcribed and biotin labeled to produce biotinylated cRNA targets according to the standard Affymetrix GeneAtlas 3′-IVT Express labeling protocol (GeneAtlas 3′ IVT Expression Kit User Manual, P/N 702833 Rev. 4, Affymetrix). Following fragmentation, 6 μg of biotinylated cRNA was hybridized for 16 h at 45 °C on the Affymetrix GeneAtlas Mouse MG-430 PM Array Strip. Strips were washed and stained using the GeneAtlas Fluidics Station according to standard Affymetrix operating procedures (GeneAtlas™ System User׳s Guide, P/N 08-0306 Rev. A January 2010). Strips were subsequently scanned using the GeneAtlas Imager system according to the standard Affymetrix protocol. Fluidics control, scan control, and data collection were performed using the GeneAtlas Instrument Control Software version 1.0.5.267. All microarray analyses were performed by the Wake Forest School of Medicine Microarray Shared Resource Core.

### 2.3 Gene set enrichment analysis (GSEA)

GSEA was performed to determine whether genes belonging to a biological pathway or a previously determined functional group were significantly overrepresented at the top or bottom of a ranked gene list compared to controls without a predefined cut-off value. This bioinformatic tool evaluates all significantly measured targets derived from a microarray experiment at the level of gene sets, which are defined based on prior biological knowledge. Thus, biologically relevant information is not missed by losing target genes due to an “arbitrarily” chosen cut-off value [4]. In this study, expression data of all 21,782 genes were compared against functional gene sets to determine whether any of these sets were enriched in GPER KO cardiomyocytes vs. intact cardiomyocytes.

## Figures and Tables

**Table 1:** Core enrichment gene list of GSEA for mitochondrial genes in female mice.

	**Gene symbol**	**Gene title**	**Rank in gene list**	**Rank metric score**	**Enrichment score**
1	HMGCS2	3-hydroxy-3-methylglutaryl-Coenzyme A synthase 2 (mitochondrial)	37	0.274	0.0158
2	MAOB	monoamine oxidase B	39	0.272	0.0332
3	COX6B2	cytochrome c oxidase subunit VIb polypeptide 2 (testis)	48	0.261	0.0495
4	HSPA1B	heat shock 70 kDa protein 1B	63	0.244	0.0645
5	UCP3	uncoupling protein 3 (mitochondrial, proton carrier)	70	0.237	0.0794
6	ALAS2	aminolevulinate, delta-, synthase 2 (sideroblastic/hypochromic anemia)	206	0.175	0.0843
7	BCKDHB	branched chain keto acid dehydrogenase E1, beta polypeptide (maple syrup urine disease)	222	0.172	0.0946
8	DUT	dUTP pyrophosphatase	311	0.159	0.1007
9	HTRA2	HtrA serine peptidase 2	313	0.158	0.1107
10	ME3	malic enzyme 3, NADP(+)-dependent, mitochondrial	431	0.145	0.1146
11	GSTZ1	glutathione transferase zeta 1 (maleylacetoacetate isomerase)	575	0.134	0.1165
12	ACOT2	acyl-CoA thioesterase 2	647	0.13	0.1215
13	PCCB	propionyl Coenzyme A carboxylase, beta polypeptide	660	0.13	0.1293
14	TIMMDC1	Translocase of inner mitochondrial membrane domain-containing protein 1	795	0.124	0.1309
15	RAF1	v-raf-1 murine leukemia viral oncogene homolog 1	851	0.121	0.1361
16	TMEM143	transmembrane protein 143	880	0.12	0.1425
17	NME4	non-metastatic cells 4, protein expressed in	920	0.119	0.1483
18	ACP6	acid phosphatase 6, lysophosphatidic	975	0.116	0.1532
19	FXN	frataxin	1014	0.114	0.1587
20	CRY1	cryptochrome 1 (photolyase-like)	1065	0.112	0.1636
21	HSD3B2	hydroxy-delta-5-steroid dehydrogenase, 3 beta- and steroid delta-isomerase 2	1079	0.112	0.1701
22	ABCB8	ATP-binding cassette, sub-family B (MDR/TAP), member 8	1234	0.107	0.1698
23	GCDH	glutaryl-Coenzyme A dehydrogenase	1258	0.107	0.1756
24	CASP7	caspase 7, apoptosis-related cysteine peptidase	1424	0.102	0.1744
25	MYL10	myosin, light chain 10, regulatory	1446	0.102	0.18
26	BCAT2	branched chain aminotransferase 2, mitochondrial	1546	0.099	0.1817
27	BZRAP1	benzodiazapine receptor (peripheral) associated protein 1	1591	0.098	0.186
28	MECR	mitochondrial trans-2-enoyl-CoA reductase	1638	0.097	0.19
29	MTIF3	Mitochondrial Translational Initiation Factor 3	1773	0.094	0.1898
30	BCL2L10	BCL2-like 10 (apoptosis facilitator)	1825	0.093	0.1934
31	ACADS	acyl-Coenzyme A dehydrogenase, C-2 to C-3 short chain	1827	0.093	0.1993
32	ECSIT	ECSIT homolog (Drosophila)	1946	0.091	0.1996
33	MRPL23	mitochondrial ribosomal protein L23	1970	0.09	0.2043
34	MRPS15	mitochondrial ribosomal protein S15	2039	0.089	0.2068
35	MRPS28	mitochondrial ribosomal protein S28	2158	0.087	0.2068
36	TBRG4	transforming growth factor beta regulator 4	2223	0.085	0.2093
37	SLC25A22	solute carrier family 25 (mitochondrial carrier: glutamate), member 22	2236	0.085	0.2142
38	MRPS11	mitochondrial ribosomal protein S11	2257	0.085	0.2187
39	BCKDHA	branched chain keto acid dehydrogenase E1, alpha polypeptide	2321	0.084	0.2211
40	TRIAP1	TP53 regulated inhibitor of apoptosis 1	2336	0.084	0.2258
41	FDXR	ferredoxin reductase	2407	0.082	0.2278
42	RHOT2	ras homolog gene family, member T2	2434	0.082	0.2319
43	MRPS24	mitochondrial ribosomal protein S24	2473	0.081	0.2353
44	MRPS35	mitochondrial ribosomal protein S35	2493	0.081	0.2396
45	MTCH1	mitochondrial carrier homolog 1 (C. elegans)	2494	0.081	0.2447
46	BCKDK	branched chain ketoacid dehydrogenase kinase	2503	0.081	0.2495
47	MRPS21	mitochondrial ribosomal protein S21	2584	0.079	0.2509
48	SHMT2	serine hydroxymethyltransferase 2 (mitochondrial)	2609	0.079	0.2548
49	PCK2	phosphoenolpyruvate carboxykinase 2 (mitochondrial)	2761	0.077	0.2527
50	CPT1A	carnitine palmitoyltransferase 1A (liver)	2776	0.076	0.2569
51	DMGDH	dimethylglycine dehydrogenase	2946	0.074	0.2538
52	MSTO1	misato homolog 1 (Drosophila)	3029	0.073	0.2546
53	DGUOK	deoxyguanosine kinase	3045	0.073	0.2586
54	PET112	glutamyl-TRNA(Gln) amidotransferase, subunit B	3058	0.072	0.2626
55	AMACR	alpha-methylacyl-CoA racemase	3069	0.072	0.2668
56	MRPS22	mitochondrial ribosomal protein S22	3103	0.072	0.2699
57	ABCF2	ATP-binding cassette, sub-family F (GCN20), member 2	3118	0.072	0.2738
58	ECI2	Enoyl-CoA Delta Isomerase 2	3129	0.072	0.2779
59	TXNRD2	thioredoxin reductase 2	3191	0.071	0.2796
60	TMEM186	Transmembrane Protein 186	3236	0.07	0.2821
61	PINK1	PTEN induced putative kinase 1	3351	0.069	0.2812
62	ALDH4A1	aldehyde dehydrogenase 4 family, member A1	3430	0.068	0.2819
63	PITRM1	pitrilysin metallopeptidase 1	3500	0.067	0.283
64	PDK4	pyruvate dehydrogenase kinase, isozyme 4	3548	0.067	0.2851
65	MIPEP	mitochondrial intermediate peptidase	3577	0.066	0.288
66	MRPL40	mitochondrial ribosomal protein L40	3589	0.066	0.2918
67	TAMM41	TAM41 Mitochondrial Translocator Assembly And Maintenance Homolog	3780	0.064	0.287
68	MSRB2	methionine sulfoxide reductase B2	3929	0.063	0.2841
69	SURF1	surfeit 1	3949	0.062	0.2872
70	ATP5G2	ATP synthase, H+ transporting, mitochondrial F0 complex, subunit C2 (subunit 9)	3962	0.062	0.2907
71	SPG7	spastic paraplegia 7, paraplegin (pure and complicated autosomal recessive)	3988	0.062	0.2935
72	BCS1L	BCS1-like (yeast)	4000	0.062	0.2969
73	ALDH5A1	aldehyde dehydrogenase 5 family, member A1 (succinate-semialdehyde dehydrogenase)	4110	0.061	0.2957
74	MARS2	methionine-tRNA synthetase 2 (mitochondrial)	4164	0.06	0.2971
75	CLN3	ceroid-lipofuscinosis, neuronal 3, juvenile (Batten, Spielmeyer-Vogt disease)	4185	0.06	0.3
76	FIS1	fission 1 (mitochondrial outer membrane) homolog (S. cerevisiae)	4205	0.06	0.303
77	POLG	polymerase (DNA directed), gamma	4255	0.059	0.3045
78	TUFM	Tu translation elongation factor, mitochondrial	4299	0.059	0.3062
79	POLG2	polymerase (DNA directed), gamma 2, accessory subunit	4333	0.059	0.3085
80	PMAIP1	phorbol-12-myristate-13-acetate-induced protein 1	4394	0.058	0.3094
81	COX11	COX11 homolog, cytochrome c oxidase assembly protein (yeast)	4427	0.058	0.3116
82	OGDH	oxoglutarate (alpha-ketoglutarate) dehydrogenase (lipoamide)	4436	0.058	0.3149
83	COX15	COX15 homolog, cytochrome c oxidase assembly protein (yeast)	4447	0.058	0.3181
84	MCAT	malonyl CoA:ACP acyltransferase (mitochondrial)	4527	0.057	0.3181
85	DECR1	2,4-dienoyl CoA reductase 1, mitochondrial	4638	0.056	0.3166
86	CASP8	caspase 8, apoptosis-related cysteine peptidase	4729	0.055	0.3159
87	TIMM17B	translocase of inner mitochondrial membrane 17 homolog B (yeast)	4752	0.055	0.3184
88	TIMM50	translocase of inner mitochondrial membrane 50 homolog (S. cerevisiae)	4791	0.055	0.3201
89	MRPS10	mitochondrial ribosomal protein S10	4848	0.054	0.321
90	AGPAT5	1-acylglycerol-3-phosphate O-acyltransferase 5 (lysophosphatidic acid acyltransferase, epsilon)	4921	0.053	0.3211
91	GBAS	glioblastoma amplified sequence	4930	0.053	0.3241
92	HSD17B10	Hydroxysteroid (17-Beta) Dehydrogenase 10	4977	0.053	0.3253
93	OXA1L	oxidase (cytochrome c) assembly 1-like	5075	0.052	0.3241
94	ETFB	electron-transfer-flavoprotein, beta polypeptide	5085	0.052	0.327
95	MRPL10	mitochondrial ribosomal protein L10	5190	0.051	0.3254
96	PHB2	prohibitin 2	5222	0.051	0.3272
97	COQ4	coenzyme Q4 homolog (S. cerevisiae)	5242	0.05	0.3295
98	SDHC	succinate dehydrogenase complex, subunit C, integral membrane protein, 15kDa	5276	0.05	0.3312
99	FDX1	ferredoxin 1	5338	0.049	0.3315
100	THG1L	tRNA-histidine guanylyltransferase 1-like (S. cerevisiae)	5342	0.049	0.3345
101	AIFM3	Apoptosis Inducing Factor, Mitochondria Associated 3	5344	0.049	0.3377
102	SLC25A15	solute carrier family 25 (mitochondrial carrier; ornithine transporter) member 15	5345	0.049	0.3408
103	PTGES2	prostaglandin E synthase 2	5367	0.049	0.343

**Table 2:** Core enrichment gene list of GSEA for mitochondrial genes in male mice.

	**Gene symbol**	**Gene title**	**Rank in gene list**	**Rank metric score**	**Enrichment score**
1	DBT	dihydrolipoamide branched chain transacylase E2	199	0.195	0.009
2	CRY1	cryptochrome 1 (photolyase-like)	255	0.181	0.023
3	BCKDHB	branched chain keto acid dehydrogenase E1, beta polypeptide (maple syrup urine disease)	440	0.151	0.028
4	MAOB	monoamine oxidase B	458	0.149	0.041
5	HMGCS2	3-hydroxy-3-methylglutaryl-Coenzyme A synthase 2 (mitochondrial)	631	0.132	0.045
6	PDK4	pyruvate dehydrogenase kinase, isozyme 4	1006	0.106	0.037
7	ABCB7	ATP-binding cassette, sub-family B (MDR/TAP), member 7	1092	0.102	0.042
8	DUT	dUTP pyrophosphatase	1286	0.095	0.042
9	METAP1D	Methionyl Aminopeptidase Type 1D (Mitochondrial)	1303	0.094	0.050
10	GSTZ1	glutathione transferase zeta 1 (maleylacetoacetate isomerase)	1346	0.092	0.057
11	UCP3	uncoupling protein 3 (mitochondrial, proton carrier)	1489	0.088	0.058
12	NR3C1	nuclear receptor subfamily 3, group C, member 1 (glucocorticoid receptor)	1533	0.087	0.064
13	MUT	methylmalonyl Coenzyme A mutase	1581	0.086	0.070
14	OPA1	optic atrophy 1 (autosomal dominant)	1820	0.079	0.066
15	NDUFS5	NADH dehydrogenase (ubiquinone) Fe-S protein 5, 15kDa (NADH-coenzyme Q reductase)	2107	0.073	0.059
16	ASAH2	N-acylsphingosine amidohydrolase (non-lysosomal ceramidase) 2	2134	0.073	0.065
17	ACOT2	acyl-CoA thioesterase 2	2138	0.073	0.071
18	MTIF2	mitochondrial translational initiation factor 2	2203	0.071	0.075
19	MSRB3	methionine sulfoxide reductase B3	2277	0.070	0.078
20	RAF1	v-raf-1 murine leukemia viral oncogene homolog 1	2334	0.069	0.082
21	GLUD1	glutamate dehydrogenase 1	2407	0.068	0.084
22	ALDH5A1	aldehyde dehydrogenase 5 family, member A1 (succinate-semialdehyde dehydrogenase)	2408	0.068	0.091
23	NLRP5	NLR Family, Pyrin Domain Containing 5	2469	0.066	0.094
24	DLD	dihydrolipoamide dehydrogenase	2485	0.066	0.099
25	PDK1	pyruvate dehydrogenase kinase, isozyme 1	2529	0.065	0.103
26	AASS	aminoadipate-semialdehyde synthase	2537	0.065	0.109
27	LDHD	lactate dehydrogenase D	2610	0.063	0.111
28	COX6B2	cytochrome c oxidase subunit VIb polypeptide 2 (testis)	2615	0.063	0.117
29	ACAT1	acetyl-Coenzyme A acetyltransferase 1 (acetoacetyl Coenzyme A thiolase)	2674	0.062	0.120
30	CLPX	ClpX caseinolytic peptidase X homolog (E. coli)	2697	0.062	0.125
31	L2HGDH	L-2-hydroxyglutarate dehydrogenase	2736	0.061	0.128
32	AIFM1	Apoptosis Inducing Factor, Mitochondria Associated 1	2820	0.060	0.130
33	SUCLA2	succinate-CoA ligase, ADP-forming, beta subunit	2846	0.060	0.134
34	HCCS	holocytochrome c synthase (cytochrome c heme-lyase)	2931	0.058	0.136
35	RHOT1	ras homolog gene family, member T1	2974	0.058	0.139
36	BCKDHA	branched chain keto acid dehydrogenase E1, alpha polypeptide	3019	0.057	0.142
37	ACADSB	acyl-Coenzyme A dehydrogenase, short/branched chain	3061	0.056	0.146
38	TRNT1	tRNA nucleotidyl transferase, CCA-adding, 1	3237	0.053	0.142
39	ABCE1	ATP-binding cassette, sub-family E (OABP), member 1	3317	0.052	0.143
40	TFB2M	transcription factor B2, mitochondrial	3417	0.051	0.143
41	GBAS	glioblastoma amplified sequence	3429	0.050	0.148
42	ETFA	electron-transfer-flavoprotein, alpha polypeptide (glutaric aciduria II)	3444	0.050	0.151
43	HTRA2	HtrA serine peptidase 2	3461	0.050	0.155
44	HSPD1	heat shock 60kDa protein 1 (chaperonin)	3504	0.049	0.158
45	POLG	polymerase (DNA directed), gamma	3642	0.048	0.156
46	GCDH	glutaryl-Coenzyme A dehydrogenase	3649	0.048	0.160
47	NIPSNAP1	nipsnap homolog 1 (C. elegans)	3769	0.046	0.159
48	TIMMDC1	Translocase of inner mitochondrial membrane domain-containing protein 1	3775	0.046	0.163
49	ACADVL	acyl-Coenzyme A dehydrogenase, very long chain	3802	0.046	0.166
50	PCCB	propionyl Coenzyme A carboxylase, beta polypeptide	3920	0.045	0.164
51	MARS2	methionine-tRNA synthetase 2 (mitochondrial)	4159	0.042	0.157
52	BID	BH3 interacting domain death agonist	4214	0.042	0.158
53	COX15	COX15 homolog, cytochrome c oxidase assembly protein (yeast)	4259	0.041	0.160
54	HADH	hydroxyacyl-Coenzyme A dehydrogenase	4307	0.041	0.162
55	DECR1	2,4-dienoyl CoA reductase 1, mitochondrial	4355	0.040	0.163
56	NDUFS1	NADH dehydrogenase (ubiquinone) Fe-S protein 1, 75kDa (NADH-coenzyme Q reductase)	4356	0.040	0.167
57	OXCT1	3-oxoacid CoA transferase 1	4385	0.040	0.169
58	BCKDK	branched chain ketoacid dehydrogenase kinase	4435	0.040	0.171
59	TRIAP1	TP53 regulated inhibitor of apoptosis 1	4508	0.039	0.171
60	PINK1	PTEN induced putative kinase 1	4586	0.038	0.171
61	MRPS28	mitochondrial ribosomal protein S28	4607	0.038	0.173
62	MRPL52	mitochondrial ribosomal protein L52	4667	0.037	0.174
63	PGS1	phosphatidylglycerophosphate synthase 1	4719	0.037	0.175
103	PTGES2	prostaglandin E synthase 2	5367	0.049	0.343

**Table 3:** Core enrichment gene list of GSEA for inflammatory response genes in female mice.

	**Gene symbol**	**Gene title**	**Rank in gene list**	**Rank metric score**	**Enrichment score**
1	MSR1	macrophage scavenger receptor 1	19680	−0.083	−0.255
2	ATP2C1	ATPase, Ca++ transporting, type 2C, member 1	19712	−0.084	−0.250
3	TLR2	toll-like receptor 2	19727	−0.084	−0.243
4	PTGER2	prostaglandin E receptor 2 (subtype EP2), 53kDa	19750	−0.085	−0.237
5	CCL22	chemokine (C-C motif) ligand 22	19779	−0.086	−0.231
6	OSMR	oncostatin M receptor	19980	−0.092	−0.232
7	C3AR1	complement component 3a receptor 1	20005	−0.092	−0.225
8	LTA	lymphotoxin alpha (TNF superfamily 1)	20034	−0.093	−0.218
9	NMI	N-myc (and STAT) interactor	20051	−0.094	−0.211
10	HPN	hepsin (transmembrane protease, serine 1)	20065	−0.094	−0.203
11	TLR3	toll-like receptor 3	20088	−0.095	−0.196
12	SLAMF1	signaling lymphocytic activation molecule family member 1	20116	−0.096	−0.189
13	EREG	epiregulin	20234	−0.100	−0.186
14	OLR1	oxidised low density lipoprotein (lectin-like) receptor 1	20359	−0.106	−0.182
15	ACVR2A	activin A receptor, type IIA	20453	−0.110	−0.177
16	DCBLD2	discoidin, CUB and LCCL domain containing 2	20543	−0.114	−0.171
17	STAB1	stabilin 1	20587	−0.117	−0.163
18	SCN1B	sodium channel, voltage-gated, type I, beta	20593	−0.118	−0.153
19	MYC	v-myc myelocytomatosis viral oncogene homolog (avian)	20604	−0.118	−0.143
20	CDKN1A	cyclin-dependent kinase inhibitor 1A (p21, Cip1)	20699	−0.124	−0.137
21	ATP2B1	ATPase, Ca^++^ transporting, plasma membrane 1	20788	−0.129	−0.130
22	CD14	CD14 molecule	20971	−0.143	−0.126
23	RNF144B	Ring Finger Protein 144B	21103	−0.157	−0.119
24	NPFFR2	neuropeptide FF receptor 2	21108	−0.158	−0.105
25	PTPRE	protein tyrosine phosphatase, receptor type, E	21116	−0.159	−0.092
26	PCDH7	BH-protocadherin (brain-heart)	21188	−0.168	−0.081
27	HIF1A	hypoxia-inducible factor 1, alpha subunit (basic helix-loop-helix transcription factor)	21349	−0.187	−0.072
28	ABI1	abl-interactor 1	21353	−0.187	−0.056
29	ABCA1	ATP-binding cassette, sub-family A (ABC1), member 1	21458	−0.212	−0.042
30	GNAI3	guanine nucleotide binding protein (G protein), alpha inhibiting activity polypeptide 3	21522	−0.225	−0.026
31	TIMP1	TIMP metallopeptidase inhibitor 1	21757	−0.434	0.001

**Table 4:** Core enrichment gene list of GSEA for inflammatory response genes in male mice.

	**Gene symbol**	**Gene title**	**Rank in gene list**	**Rank metric score**	**Enrichment score**
1	TIMP1	TIMP metallopeptidase inhibitor 1	3	0.804	0.061
2	CD48	CD48 molecule	30	0.397	0.091
3	CD14	CD14 molecule	59	0.322	0.114
4	SCN1B	sodium channel, voltage-gated, type I, beta	76	0.303	0.136
5	CYBB	cytochrome b-245, beta polypeptide (chronic granulomatous disease)	96	0.273	0.156
6	CDKN1A	cyclin-dependent kinase inhibitor 1A (p21, Cip1)	159	0.227	0.171
7	OSMR	oncostatin M receptor	163	0.223	0.188
8	C3AR1	complement component 3a receptor 1	215	0.200	0.201
9	CCL7	chemokine (C-C motif) ligand 7	246	0.188	0.214
10	ICAM4	intercellular adhesion molecule 4 (Landsteiner-Wiener blood group)	269	0.182	0.227
11	MYC	v-myc myelocytomatosis viral oncogene homolog (avian)	300	0.175	0.238
12	EMR1	egf-like module containing, mucin-like, hormone receptor-like 1	332	0.167	0.250
13	GCH1	GTP cyclohydrolase 1 (dopa-responsive dystonia)	452	0.148	0.256
14	TLR2	toll-like receptor 2	460	0.147	0.267
15	CCL2	chemokine (C-C motif) ligand 2	529	0.139	0.274
16	IL18	interleukin 18 (interferon-gamma-inducing factor)	569	0.136	0.283
17	EMP3	epithelial membrane protein 3	627	0.132	0.290
18	PTPRE	protein tyrosine phosphatase, receptor type, E	654	0.130	0.299
19	EREG	epiregulin	695	0.128	0.307
20	STAB1	stabilin 1	870	0.117	0.308
21	FZD5	frizzled homolog 5 (Drosophila)	891	0.117	0.316
22	RHOG	ras homolog gene family, member G (rho G)	968	0.112	0.321
23	SERPINE1	serpin peptidase inhibitor, clade E, member 1	1019	0.110	0.327
24	SRI	sorcin	1079	0.108	0.332
25	SLC7A2	solute carrier family 7 (cationic amino acid transporter, y+ system), member 2	1090	0.108	0.340
26	CCL24	chemokine (C-C motif) ligand 24	1246	0.102	0.341
27	LIF	leukemia inhibitory factor (cholinergic differentiation factor)	1273	0.101	0.347
28	SEMA4D	sema domain, immunoglobulin domain (Ig), transmembrane domain (TM) and short cytoplasmic domain, (semaphorin) 4D	1374	0.099	0.350
29	CMKLR1	chemokine-like receptor 1	1397	0.098	0.357
30	P2RX4	purinergic receptor P2X, ligand-gated ion channel, 4	1426	0.097	0.363
31	SLC31A1	solute carrier family 31 (copper transporters), member 1	1443	0.097	0.370
32	IL6	interleukin 6 (interferon, beta 2)	1634	0.092	0.368
33	CHST2	carbohydrate (N-acetylglucosamine-6-O) sulfotransferase 2	1747	0.089	0.369
34	ITGA5	integrin, alpha 5 (fibronectin receptor, alpha polypeptide)	1890	0.086	0.369
35	HBEGF	heparin-binding EGF-like growth factor	1894	0.086	0.376
36	CX3CL1	chemokine (C-X3-C motif) ligand 1	1920	0.086	0.381

## Appendix A Supplementary data

Application 1

Application 2
